# Does investment in palm oil trade alleviate smallholders from poverty in Africa? Investigating profitability from a biodiversity hotspot, Cameroon

**DOI:** 10.1371/journal.pone.0256498

**Published:** 2021-09-01

**Authors:** Lacour M. Ayompe, Raymond N. Nkongho, Cargele Masso, Benis N. Egoh

**Affiliations:** 1 Department of Earth System Science, University of California Irvine, Irvine, California, United States of America; 2 International Institute for Tropical Agriculture (IITA) Cameroon, Yaoundé, Cameroon; 3 Department of Agronomic and Applied Molecular Sciences, University of Buea, Buea, Cameroon; Szechenyi Istvan University: Szechenyi Istvan Egyetem, HUNGARY

## Abstract

In this study we investigate whether the increasing investment in smallholder oil palm plantations that contributes to deforestation is motivated by financial gains or other factors. We evaluate the financial viability of smallholder farmers selling fresh fruit bunches (FFBs) to intermediaries or agro-industrial companies with mills, or processing the FFBs in artisanal mills to produce palm oil. We use data collected in four oil palm production basins in Cameroon and carried out a life cycle assessment of oil palm cultivation and CPO production to understand financial gains. We use payback period (PBP), internal rate of return (IRR), benefit cost ratio (BCR) and net present value (NPV) for 1 ha of oil palm plantation over 28 years at a base discount rate of 8% to asses viability. Our results show that smallholders make more money processing their FFBs in artisanal mills to produce CPO than selling FFBs to intermediaries or agro-industrial companies with mills. The sensitivity analysis show that land ownership is the single most important parameter in the profitability of investment in palm oil cultivation and trade. In addition to land cost, smallholders suffer from borrowing at high interest rates, high field management costs, while recording low on-farm FFB/processing yields. To improve the financial viability of smallholders investing in oil palm cultivation, measures are needed to encourage them to access land, get loans at reduced interest rates, reduce the cost of field management, adopt good agricultural practices to improve on-farm FFB/processing yields, as well as to generate additional revenue from the sale of other products.

## 1. Introduction

Palm oil is an edible oil with a high yield, various economic benefits, and many diverse uses [1]. Global demand for palm oil has been on the increase because of its many useful applications in the food, cosmetics and bio-fuel industries [2]. Global production of crude palm oil (CPO) is estimated at 74 Mt, or 36% of the total world vegetable oil production in 2019 compared to soybean (56 Mt or 27%) and rapeseed oil (28 Mt or 14%) [3]. Although oil palm originated in Africa, the African continent has not really taken advantage of the increasing global demand for palm oil. In 2014, about 51.2 Mt (89.3%) of CPO was produced in Asia, 3.3 Mt (5.8%) in Latin America and 2.8 Mt (4.9%) in Africa. Cameroon ranked 13^th^ globally with 253,000 tons of CPO produced in 2019 [4] with about 170,000 ha cultivated by 2017. Indeed, between 2000 and 2017 Cameroon expanded its oil palm cultivated area by 114,094 ha to become the world’s 7^th^ largest oil palm fruit producer with annual production of about 3,110,296 tons second to Nigeria with 7,759,426 tons and ahead of Ghana and Cote d’Ivoire with 2,469,763 and 2,227,000 tons respectively. During this time, the average annual change in oil palm fruit area harvested increased from 1.5%/yr between 1980 and 2000 to 12.0%/yr [4]. This increase in area cultivated is attributed to an increase in smallholders because no new oil palm plantations were established by agro-industrial companies during this period. The increases in area cultivated with oil palm is not unique to Cameroon. Increasing global demand for CPO and its products has also been met by a rapid expansion of oil palm plantations in other countries, especially in Malaysia and Indonesia [5] at the expense of forest areas creating environmental concerns [6]. Continuous production of fresh fruit bunches (FFBs) in oil palm plantations and milling them to produce CPO has been essential to meet growing demand for palm oil [7]. Governments of developing and emerging countries in all tropical regions increasingly promote oil palm cultivation as a major contributor to poverty alleviation, as well as food and energy independence [8].

Smallholder agriculture has long served as the dominant economic activity for people in sub-Saharan Africa, and it will remain enormously important for the foreseeable future [9]. In Nigeria and Ethiopia, smallholder agriculture has played an important role in enhancing sustainable development and improving food security [10, 11]. However, the expansion of oil palm plantations by smallholder farmers has not happened without negative impacts on the environment. In a recent study, [12] reported that 73% of smallholder farmers in Cameroon clear natural forest to expand their oil palm plantations. Also, 67% of oil palm expansion from 2000–2015 in the South West Region of Cameroon occurred at the expense of forest. The expansion and deforestation carried out by non-industrial producers has been occurring near low-efficiency informal mills, unconstrained by the location of high-efficiency agro-industrial mills [13]. These reveal that non-industrial oil palm growers increasingly play a disproportionate role in deforestation, many of which are engaged in informal supply chains through the use of non-industrial mills. Indeed, smallholders have been characterized as the weakest link in the oil palm plantations sector due to poor practices and low yields compared to agro-industrial companies [14]. Several of the environmental impacts could be avoided with less expansion to the forest if smallholders improved their yields. Although smallholders increasingly use high-yielding seedlings, their oil palm plantations average 7.7 tons of FFBs/ha/yr well below the potential 20 tons of FFBs/ha/yr for Cameroon [12]. It is worth noting that Cameroon’s high oil palm fruit yield is mainly driven by private agro-industrial oil palm plantations. The low FFB yields is not unique to smallholder farmers in Cameroon. In Ghana, Rhebergen et al. [15] reported that smallholder FFB yields average 7 t/ha/yr.

Oil palm is a native of the central African region, and has been cultivated in Cameroon since times immemorial to produce cooking oil, palm wine, and soap [16]. 80% of Cameroonians consume red palm oil with an estimated 30% produced by artisanal mills [17]. Both palm oil and kernel oil are used as food (about 90%) and red palm oil is sold to soap production and oil refining companies. Other uses include the pressed cake of palm kernel which is a good source of livestock feed while other parts are used for roofing and fencing [18, 19]. In addition, waste like empty fruit bunches are good sources of organic fertilizer and a suitable substrate for mushroom (*Agaricus* spp.) cultivation. These variety of uses which also include mill effluent as a source of bio-energy has created a huge demand for oil palm cultivation in the country [20]. Aside from the uses, oil palm plantation development offers direct and indirect employment opportunities to the local population as well as income through payment of taxes to the state [21].

The demand for palm oil and its contribution in job creation coupled with the fall in market prices for alternative crops such as cocoa and coffee in the late 1980s and early 1990s, including the devaluation of the Franc CFA, caused farmers to divert to the cultivation of oil palm [17]. This created a new investment opportunity for richer independent smallholders who could afford to develop larger areas of oil palm plantations [22]. Interestingly, many of these smallholders made this switch and some continue to invest in oil palms without necessarily assessing the financial gains from their investment. Indeed, it is not clear if smallholders are investing in oil palm cultivation because of government incentives, prestige of owning a farm or because of the financial benefits. Cameroon has been a net importer of CPO for several decades and the government wants to increase palm oil production so it has been investing in smallholder schemes to achieve this objective. Nevertheless, Cameroon is seen as having one of the fastest rates of deforestation, high biodiversity and high levels of poverty. It is therefore the perfect place to understand what is driving the current expansion by smallholders in Africa.

Financial viability assessments enable smallholders identify suitable conditions under which they will be able to generate sufficient return on their investment to improve their livelihoods while minimizing environmental impacts. Therefore, the aim of this study is to understand the extent to which financial gains influence smallholder farmers’ decision to invest in oil palm cultivation and CPO production. We also explore how smallholders could improve on profitability to reduce expansion of their farms. We achieve this by 1) developing a financial costs and benefits discounted cash flow model over an oil palm plantation’s life of 28 years (25 years after the first harvest); 2) analyzing the financial viability of establishing smallholder oil palm plantations in different production basins in Cameroon; 3) assessing the profitability from selling the FFBs to intermediaries or agro-industrial companies with mills or processing the FFBs in artisanal mills to produce CPO; 4) assessing the sensitivity of different inputs on the financial viability of investing in smallholder oil palm cultivation and CPO production in artisanal mills.

## 2. History of the oil palm in Cameroon

**Fig 1:**
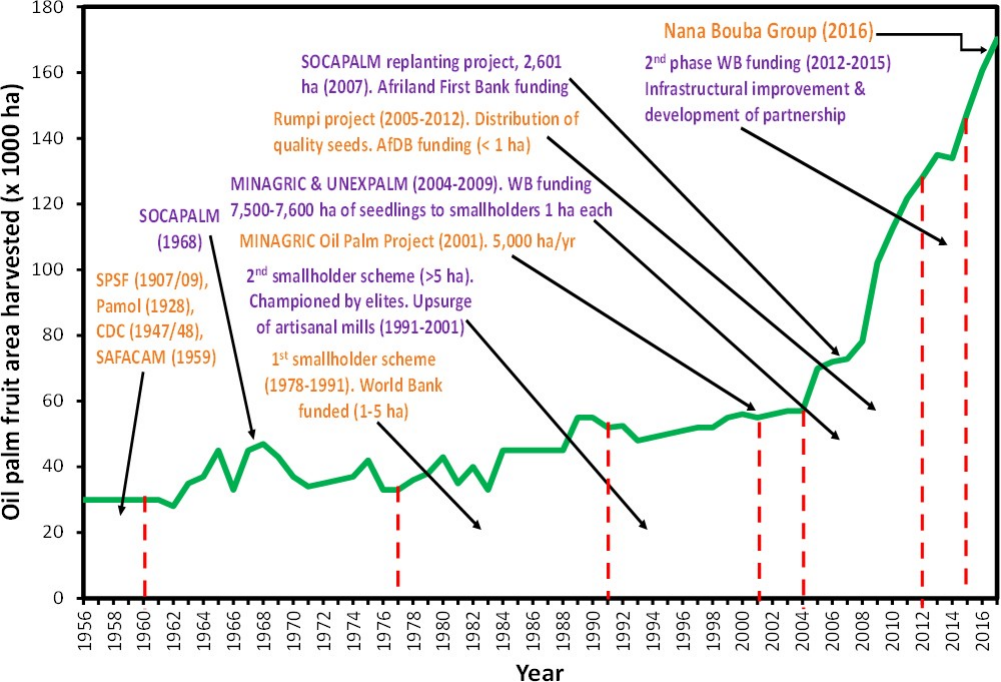
Growth in oil palm fruit area cultivated and smallholder support schemes in Cameroon. (Source: [4, 20]).

Major expansion of oil palm plantations in Cameroon started in the 1900s through large-scale, state-owned corporations and later on private companies (Fig 1). Industrialization of oil palm plantations in Cameroon began with the establishment of five large private and public agro-industrial oil palm plantations (Société des Palmeraies de la Ferme Suisse (SPFS, 1907/1909), Paul-Andrew-Martin-Oliver-Lawrence (Pamol, 1928), Cameroon Development Corporation (CDC, 1947/1948), Société Africaine Forestière et Agricole du Cameroun (SAFACAM, 1959), Société Camerounaise de Palmeraies (SOCAPALM, 1968)) [23, 24]. The latest large oil palm plantation is Grenfield that was established in 2016 by the Nana Bouba Group. During the last five decades after the establishment of the first 5 agro-industrial oil palm plantations, smallholder oil palm farming in Cameroon was encouraged and supported through specific government programs and private initiatives.

In the late 1970s, the government of Cameroon as part of its poverty alleviation strategy developed the first smallholder oil palm scheme. It was funded by the World Bank and implemented by Fonds National de Développement Rurale (FONADER) between 1977 and 1991. The scheme encouraged smallholders to establish 1–5 ha of oil palm plantations around nucleus palm oil mills [25]. About 35,000 ha of smallholdings were developed during the FONADER-sponsored scheme [24]. In addition, between 1991 and 2001 many elites (people of high social status in their village or clan of origin) established oil palm plantations larger than 5 ha. Most of the oil palm plantations established by elites were not in proximity to large agro-industrial palm oil mills and this resulted in the upsurge of artisanal mills. Some government and privately sponsored projects aimed at increasing palm oil production through smallholders after 2001 include: the Ministry of Agriculture’s (MINAGRIC) Oil Palm Project launched in 2001 aimed at planting 5,000 ha/year [24]; MINAGRIC and UNEXPALM (Union of Oil Palm Growers) project (2004–2009) that offered 1 ha of oil palm seedlings to 7,600 smallholder farmers; the Rumpi project (2005–2012) funded by the African Development Bank that distributed 1 ha of quality seeds to each smallholder farmer; SOCAPALM’s smallholder replanting project covering 2,600 ha in 2007; and the 2^nd^ phase of World Bank funding (2012–2015) for infrastructural improvement and development of partnerships.

The Government of Cameroon (GoC) has identified oil palm as one of those crops that could greatly uplift people out of poverty as part of its poverty reduction strategy by 2035. The government has therefore supported and funded smallholder farmers as described above. Despite the problems plaguing the oil palm sector, the GoC is yet to come up with a clear and convincing strategy for sustainable production of palm oil in the country. However, on July 2013, a ministerial decision (No. 00250/CAB/MINADER/29) created an inter-ministerial committee charged with developing a National Palm Oil Strategy. Other recent initiatives by the GoC to develop the palm oil sector include the establishment of the Agricultural Value Chain Development Project in 2015 (with palm oil, plantain and pineapple as target commodities), and the National palm oil and rubber development project. In 2018 Cameroon became a partner and signatory to the Tropical Forest Alliance (TFA2020) and developed principles for sustainable oil palm development. It therefore subscribed to the TFA-Africa Palm Oil Initiative (TFA-APOI) which aims to reduce tropical deforestation and create a thriving palm oil industry that brings jobs and wealth to local communities in a way that is socially and environmentally sustainable and that protects tropical forests in Africa. In June 2021, TFA in collaboration with IDH, WWF, Proforest and under the supervision of the Ministry of the Environment, Nature Protection and Sustainable Development (MINEPDED), organized a national dialogue of stakeholders from the cocoa, oil palm, rubber, banana and timber sector on how sustainability efforts can be strengthened within a common platform. Though these initiatives are underway, including the development of a national oil palm program, it remains to be seen how they will translate into sustainable palm oil production on the ground.

## 3. Materials and methods

The Roundtable on Sustainable Palm Oil (RSPO) defines smallholders as farmers growing oil palm, sometimes along with subsistence production of other crops, where the planted areas of oil palm are usually below 50 ha in size. The farm provides the majority of income to the family, and in turn the family provides the majority of labor on their farm. Smallholders can be organized as either “scheme” or “independent” smallholders [26]. We adopt this definition, although in Cameroon, some smallholders are elites who live and work in big cities but appoint a family member who plays a major role in the responsibilities on the farm.

The financial costs and potential revenues from the cultivation and sale of oil palm FFBs to intermediaries or agro-industrial mills or sale of CPO produced by processing FFBs in artisanal mills depend on a number of economic and geographic factors. We develop a spreadsheet financial model in Microsoft Excel 2019 to assess the financial viability of smallholders selling their FFBs to either intermediaries or agro-industrial mills or CPO processed in artisanal mills in four production basins in Cameroon. The model assesses different financial metrics (payback period (PP), net present value (NPV), internal rate of return (IRR), and benefit cost ratio (BCR)) for 1 ha of oil palm plantation, over a period of 28 years, discounts future costs and benefits to present value to account for the time value of money and finally investigates how sensitive the NPV is to changes of key input values.

### 3.1 Description of study areas

Cameroon has a total surface area of 475,400 km^2^ of which 185,960 km^2^ (about 39%) was forest area in 2016 [27]. By the end of 2017, about 1% of the forest area was used for oil palm cultivation. The climate is tropical, semi-arid in the north, and humid and rainy in the rest of the country. On the coast, the average annual rainfall ranges between 2,500 and 4,000 mm while it is between 1,500 and 2,500 mm in the inland South. Cameroon is endowed with rich natural resources, including oil and gas, minerals, and high value species of timber. Its economy depends strongly on agricultural products, such as coffee, cotton, cocoa, maize and cassava. The poverty reduction rate is lagging behind its population growth rate. The number of poor people living below $1.9/day in Cameroon increased by 12% to 8.1 million (about 40% of population) between 2007 and 2014 with 56% of them living in the Northern regions [28]. Cameroon has 22 million ha of tropical forests which provide an important source of revenue, employment and contributes to the provision of a variety of ecosystem services necessary for the livelihood of people in the country. Cameroon is very rich in biodiversity and provides habitat for over 9,000 plant species, 910 bird species, and 320 mammal species. Parts of Cameroon, including some of the oil palm basin such as the South West region is part of the Guinian Forests Biodiversity Hotspot designated by Conservation International and some smaller areas have been identified as Key Biodiversity Areas [29]. Indeed, most of the areas suitable for oil palm cultivation in Cameroon, also overlap with areas of high primate vulnerability in the country [30]. In addition to oil palm cultivation that threatens Cameroon’s rich biodiversity, about 40% of the national forest area is used for timber extraction, while 20% is covered by national parks, forest reserves, and hunting zones [31].

### 3.2 Data

We used data from two sources including the National Institute of Statistics [32] and primary/secondary data collected by Nkongho [20] in four oil palm production basins in Cameroon. According to Nkongho [20], the data was collected during face to face interviews with smallholder farmers and oil palm mill owners. The data relates to quantities and costs of all inputs and outputs of the establishment, maintenance, harvesting and sales of palm oil. Future amounts of inputs and outputs are estimated. The US dollar to Cameroon FCFA exchange rate was assumed to be $1:550 FCFA. CPO production was assumed to be 173 l/t FFBs during the peak season and 158 l/t FFBs during the low season. Field management costs were assumed to vary between years 0 and 3 as shown in Table 1. Where smallholders do not have customary rights to land, we use the land cost in Table 2 to investigate the effect of land cost on financial profitability in different locations. We also use the FFB sale price to intermediaries and agro-industries, palm oil processing cost and palm oil sale prices in Table 2 in our analysis.

**Table 1:** Basic data common to all locations. (Source: Nkongho [20]).

Parameter description	Unit	Value
Discount rate	%	8
Oil palm useful life	Years	25
FFB selling price	$/t	71–85
Exchange rate	FCFA/$	550
Peak season FFB production ratio	%	65
Low season FFB production ratio	%	35
Peak season CPO production	l/t FFB	173
Low season CPO production	l/t FFB	158
Field management cost (year 0)	$/ha	842
Field management cost (years 1&2)	$/ha	282
Field management cost (year 3)	$/ha	329

Note: l/t FFB is liters per ton of fresh fruit bunch; $/t is dollars per ton; $/ha is dollars per hectare

**Table 2:** Economic data in different locations. (Source: Nkongho [20]).

Description	Dibombari	Eseka	Muyuka	Lobe
Minimum land cost ($/ha)	609	636	1,364	364
Maximum land cost ($/ha)	909	1,000	4,000	727
Peak season FFB sale price to intermediary ($/t)	76	73	75	67
Low season FFB sale price to intermediary ($/t)	91	82	86	74
FFB sale price to agro-industry ($/t)	87	87	91	76
Palm oil processing cost ($/t)	49	50	38	43
Peak season palm oil sale price ($/l)	0.91	0.73	0.93	0.73
Low season palm oil sale price ($/l)	1.22	0.99	1.20	0.92

Capital costs are expenses that occur in year 0 and include the cost of acquiring land, forest clearing, felling and burning, nursery, transportation, planting, and fertilizer. Land cost varies in different production basins and is highest in Muyuka (4.2905°N, 9.4145°E) and lowest in Lobe (4.9479°N, 8.8724°E) (Table 2). The field management cost in year 0 (842 $/ha) consists of forest clearing (64 $/ha), felling and burning (91 $/ha), nursery (527 $/ha), transportation (36 $/ha), planting (91 $/ha), fertilizer (33 $/ha). In cases where smallholders have custodian rights over the land, estimated oil palm plantation development costs range from 1,550–1,740 $/ha. The lower value accounts for replanting on an existing oil palm plantation, while the higher value is for establishing a new oil palm plantation on forested land. When smallholders have to purchase land, their development costs range from 2,344–2,644 $/ha in Dibombari (4.1761°N, 9.6592°E), 2,371–2,735 $/ha in Eseka (3.6427°N, 10.7831°E), 3,099–5,735 $/ha in Muyuka and 2,099–2,462 $/ha in Lobe. Total development costs of 2,000–3,500 $/ha including land cost have been reported in studies by Cramb and Ferraro [33] and Budidarsono et al. [34]. Recurrent costs are essentially field management costs that occur between years 1 and the end of the economic life of the oil palm plantation. These include the cost for forest under-brushing/slashing, fertilizer purchase and application, circle weeding, harvesting and transportation of FFBs.

### 3.3 Financial analysis

The first objective was to develop a spreadsheet model to describe the revenues and costs associated with a smallholder who either sells his FFBs to an intermediary or agro-industrial company with mill or sells CPO from FFBs processed in his artisanal mill over the oil palm plantations useful life. We consider a base case where the smallholders had customary land rights and therefore do not have to purchase land. We assume a discount rate of 8% and average annual yield of 8.4 tons of FFBs per ha per year during the period when the oil palm plantation produces FFBs. We also assume that 65% of FFBs are produced during the peak season and 35% during the low season over a useful life of 25 years after the first harvest. For a given year t, we evaluate the net cash flow (CF_t_ in $) as the revenue (R_t_ in $) minus the recurrent costs (C_t_ in $) (Eq 1):

$${\text{CF}}_{\text{t}}={\text{R}}_{\text{t}}-{\text{C}}_{\text{t}}$$ (1)

The cumulative cash flow (CCF) for a given year (t) is obtained by summing the net annual cash flow for the previous years with that of the year of interest. It is obtained as:

$${\text{CCF}}_{\text{t}}={-\text{C}}_{0}+\sum_{\text{t}=1}^{\text{t}=\text{T}}\left({\text{R}}_{\text{t}}{-\text{C}}_{\text{t}}\right)$$ (2)

A description of the metrics (NPV, IRR, BCR and PBP) used to evaluate the financial viability of smallholders investing in oil palm cultivation and the equations employed to calculate them are detailed in (S1 File).

### 3.4 Sensitivity analysis

To investigate the extent to which variations in our input data affects smallholder’s decision to invest, we use sensitivity analysis. It consists of identifying parameters where small variations in the input data would result in a change in the farmer’s decision to invest in an oil palm plantation and either sell their FFBs or mill them in artisanal mills. Newnan et al. [35] describe sensitivity analysis as the process of better evaluating the variation of any given estimate that is required to change a particular decision.

Sensitivity analysis enables us to evaluate financial risks especially when factors affecting financial viability change in the negative direction. From a financial standpoint, we investigate how strong is the financial viability for a smallholder to invest in oil palm cultivation with varying field and marketplace conditions. We investigate the effect of changes in discount rate, FFB yield, field management cost, CPO processing cost, and land cost on NPV. Our sensitivity analysis consists of evaluating the NPV assuming defined percentages or absolute changes in the relevant inputs, considered singly. Comparing the resulting changes in the values of the NPV gives insights into how sensitive it is for smallholders investing in oil palm plantations and selling their FFBs or processing them in artisanal mills to produce CPO is to changes in each of the factors considered.

## 4. Results

### 4.1 Financial analysis

**Fig 2:**
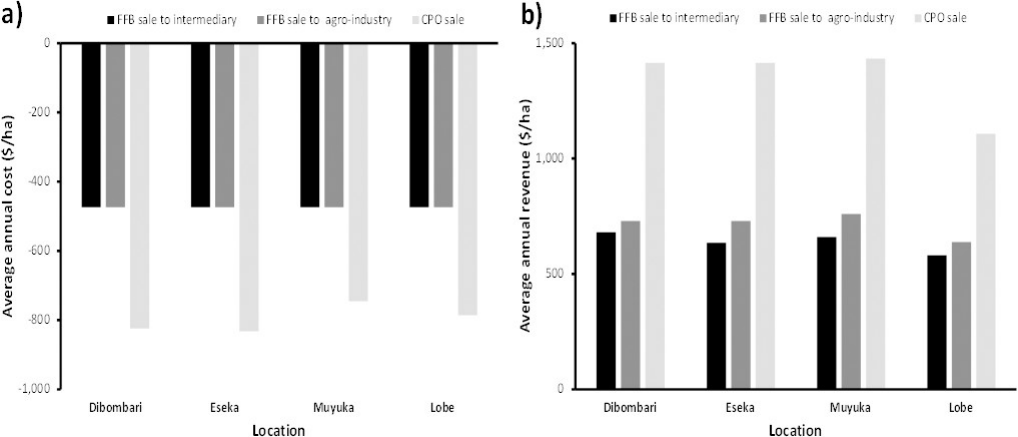
a) Average annual cost, b) average annual revenue in Dibombari, Eseka, Muyuka and Lobe.

**Fig 3:**
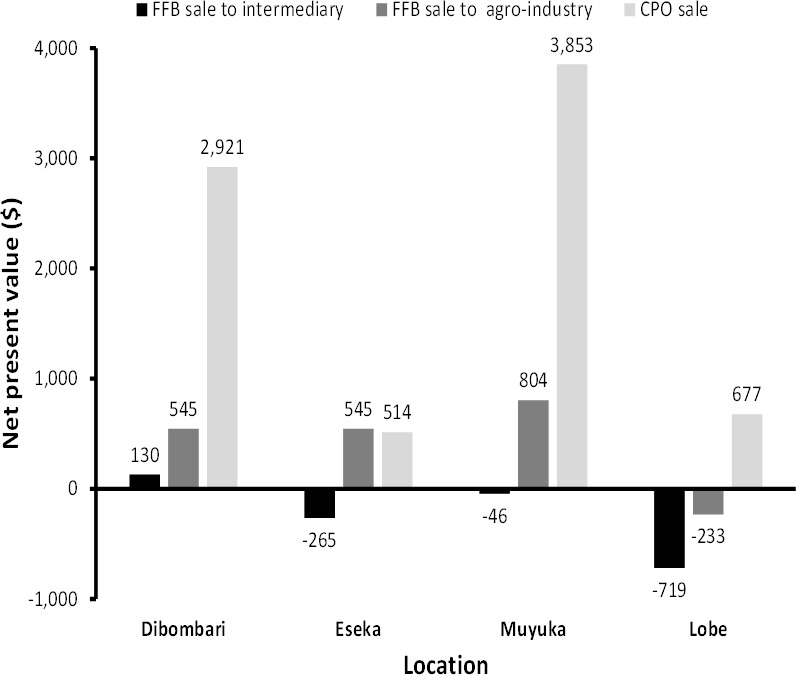
NPV in different production basins.

The results show that smallholders on average spend more annually when they produce and sell CPO than selling only FFBs (Fig 2A). Nevertheless, they receive more average annual revenue when they produce and sell CPO than FFBs (Fig 2B). The average annual revenue for producing and selling FFBs to intermediaries is almost the same as selling to agro-industries in all four locations but with a slight increase in revenue when selling to agro-industries. In all 4 locations the average annual cost over the life of the oil palm plantation for producing FFBs and selling same to either intermediaries or agro-industrial companies with mills is 474 $/ha. When looking at the different locations both the cost and revenue for producing and selling FFBs or CPO are almost the same in all locations except for Muyuka and Lobe that have a slightly lower cost and revenue for producing and selling CPO respectively. In general, from our NPV analysis it is more profitable to sell CPO than to sell FFBs except for Eseka where there is almost no difference between selling FFBs or CPO (Fig 3). In addition, it is better to sell FFBs to agro-industries than to sell to intermediaries. Interestingly, selling FFBs to either intermediaries or agro-industries in Lobe is not profitable. This is also the case with selling FFBs to intermediaries in Eseka and Muyuka.

Assessing all 4 locations, our results show that it is more profitable to invest in oil palms in Dibombari and Muyuka than in Eseka and Lobe (Table 3). Accordingly, both Dibombari and Muyuka have the shortest payback periods, highest benefit cost ratio and internal rate of return (19.4% and 22.0% respectively). Even in both Eseka and Lobe where payback periods are highest (12.9 and 15.9 years respectively) one could still make profits with IRR above 10%. The payback periods are lowest in Muyuka (6.9 years) and Dibombari (7.3 years) when the smallholders sell the CPO produced as a result of using artisanal mills. The benefit cost ratio is highest in Muyuka (1.5) when smallholders sell CPO and lowest in Lobe (0.9) when they sell FFBs to intermediaries. The IRR is highest in Muyuka when smallholders sell CPO while it is lowest in Lobe when they sell FFBs to intermediaries. The results show that Dibombari and Muyuka have better economic returns partly because farmers have access to better processing facilities and the production basins are close to major markets.

**Table 3:** Payback period, benefit cost ratio and internal rate of return in different locations.

	Payback period (years)			BCR			IRR (%)		
*Location*	*FFB(i)*	*FFB(a)*	*CPO*	*FFB(i)*	*FFB(a)*	*CPO*	*FFB(i)*	*FFB(a)*	*CPO*
Dibombari	11.4	10.2	7.3	1.0	1.1	1.3	8.7	10.8	19.4
Eseka	12.9	10.2	10.2	1.0	1.1	1.1	6.4	10.8	10.6
Muyuka	12.0	9.6	6.9	1.0	1.1	1.5	7.7	12.0	22.0
Lobe	15.9	12.7	9.9	0.9	1.0	1.1	3.0	6.6	11.4

FFB(i): FFB sale to intermediaries; FFB(a): FFB sale to agro-industrial mills; CPO: Crude palm oil sale

### 4.2 Sensitivity analysis

#### 4.2.1 Effect of discount rate

**Fig 4:**
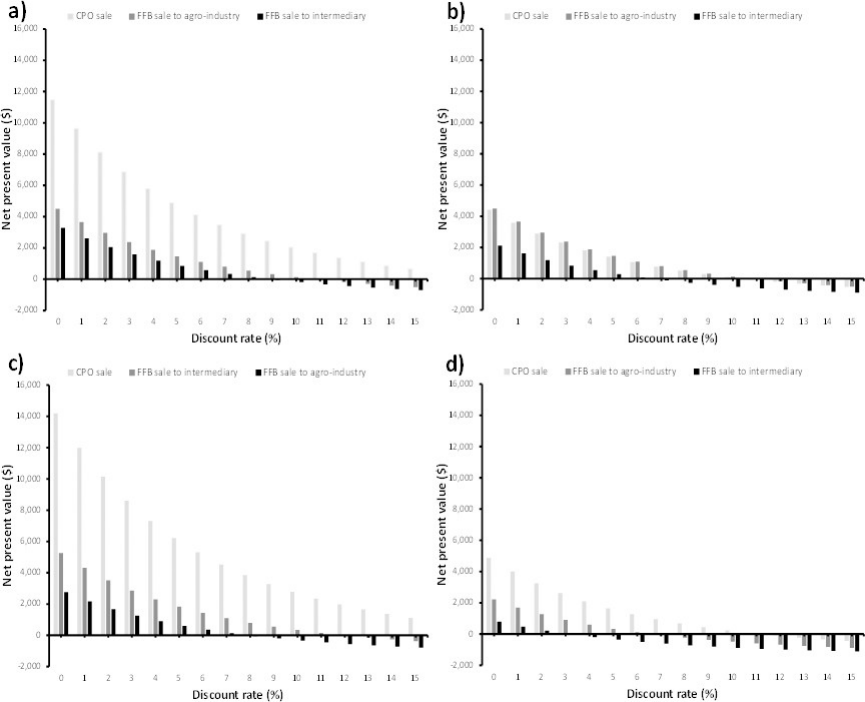
Variation of net present value with discount rate a) Dibombari, b) Eseka, c) Muyuka, d) Lobe.

The sensitivity analysis results show that financial viability increases as the discount rate decreases (Fig 4A–4D). Indeed, in both Dibombari and Muyuka, smallholders could make between $5,000 and $14,000 when they borrow money with a discount of 0–7%. However, the profitability decreases significantly around about 9% discount rate. However, in both Eseka and Muyuka, the profit margins are small with a maximum of $5,000 when the borrowing rate is 5% or less. In these 2 regions, smallholders stand a bigger chance of losing money with a discount rate of 6% or higher. These results also reiterate the fact that it is more viable to sell CPO than FFB especially if the borrowing rate is <8% except for Eseka where there is not much difference. Investing in oil palm cultivation in Lobe does not look viable as margins are very small and even negative after 4% discounted rate.

#### 4.2.2 Effect of land cost

**Fig 5:**
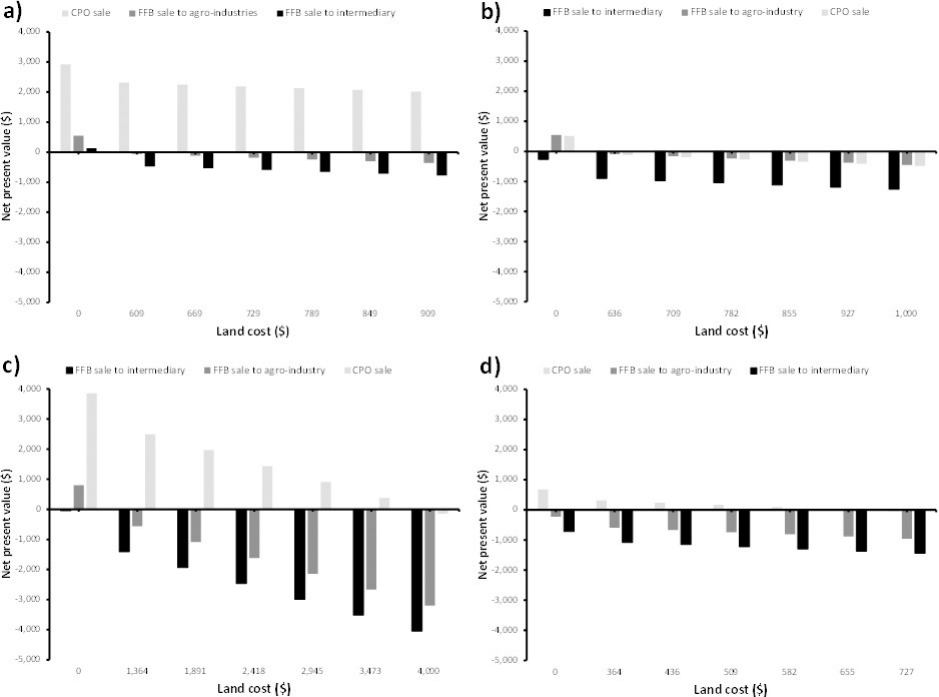
Variation of net present value with land cost a) Dibombari, b) Eseka, c) Muyuka, d) Lobe.

Land is an important resource that impacts severely on the profitability of oil palm investments. Most oil palm smallholders do not have to buy land when they have customary rights, although land ownership in Cameroon is one of “*mise en valeur*” (own due to enhancement or use). However, investors who do not have customary rights have to buy land as part of the initial investment cost. A key finding is that in all locations, investment in palm oil production is mostly profitable if land is not bought (Fig 5). However, the buying of land makes the investment profitable only in 2 locations and mostly when smallholders produce and sell CPO instead of FFBs. The severity of the impact of land cost is seen particularly in Lobe where there are loses as soon as you include land cost except for very small gains at less than $500/ha. These results are of importance because most investors who do not have customary rights buy land when establishing oil palm plantations. Even in places where smallholders can still make money after they have bought land as part of the initial investment, the profit margins are very small and only up to $4,000/ha. In Muyuka, if land cost is in the range of 1,364–3,473 $/ha and smallholders mill their FFBs and sell the CPO, they can make up to $4,000 only (Fig 5C). Land cost therefore plays a major role in the viability of investment in oil palm plantations in many locations.

#### 4.2.3 Effect of field management, FFB yield and CPO processing cost

**Fig 6:**
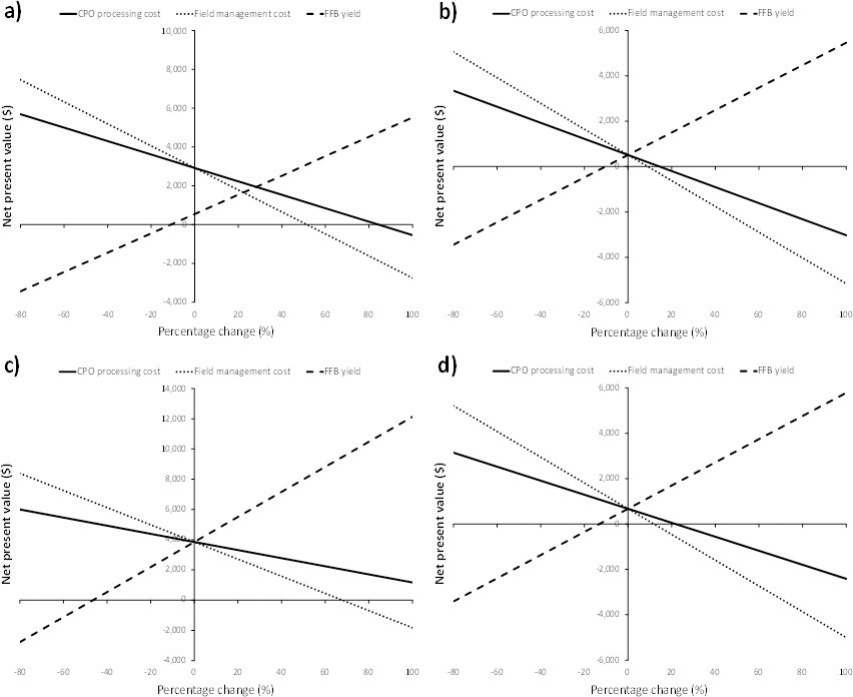
NPV against changes in CPO processing cost, field management cost and FFB yield a) Dibombari, b) Eseka, c) Muyuka, d) Lobe.

Results of the sensitivity analysis also show that increases in field management and CPO processing costs negatively affect the financial viability of smallholders who process their FFBs in artisanal mills and sell the CPO. In contrast, increases in FFB yield positively affect financial viability (Fig 6A–6D). The shallowness of the slopes shows that changes in CPO processing cost have the least impact on financial viability. In Dibombari, it is financially viable for smallholders if the average annual CPO processing cost is ≤730 $/ton; the average annual field management cost is ≤664 $/ha; and the average annual FFB yield is ≥6.7 ton/ha. In Lobe, it is financially viable for smallholders if the average annual CPO processing cost is ≤434 $/ton; the average annual field management cost is ≤474 $/ha; and the average annual FFB yield is ≥8.4 ton/ha. The above only account for external labor costs and do not include compensation to the smallholders for their time invested in field maintenance and CPO processing.

## 5. Discussion

Although concentrated in Southeast Asia, oil palm production expanded by 1.2 million ha in sub-Saharan Africa between 1990 and 2017, with expansion accelerating in several heavily forested countries since 2000 [36]. Recently, Ordway et al. [13] showed that contrary to the publicized narrative of industrial-scale expansion, most oil palm expansion and associated deforestation is occurring outside large agro-industrial concessions with about 67% of oil palm expansion occurring at the expense of forest in Southwest Cameroon. In this study, our aim was to understand why smallholder farmers, now heavily implicated in deforestation are investing in oil palm plantations and how these investments can be sustainable. The financial assessments indicate that investment in oil palm plantations is mostly profitable in all 4 regions although profit margins may be small in places like Lobe. One of the key factors that determine profitability of the investment is milling of the products as smallholders could make up to $14,000 if they sell CPO rather than FFBs. Except in Eseka, smallholders generally have the highest return on investment when they process their FFBs in artisanal mills and sell the CPO compared to selling FFBs to either intermediaries or agro-industrial mills. This is not surprising as recent studies show that there has been a proliferation of artisanal mills in these production basins [14, 36] despite their low extraction rates (13–15%) compared to agro-industrial mills (21–23%) [37]. Indeed, smallholders could make a profit of between 19–22% in Dibombari and Muyuka if they sell CPO and they could pay back their initial investment within 7 years. Nevertheless, some smallholders decide to sell their FFBs when they do not own or have easy access to artisanal mills or are not in close proximity of agro-industrial mills.

Our results also reveal that the average net annual income gained by smallholders is about $545 per ha. Therefore, smallholders who own 5–10 ha of oil palm plantations would have average net annual revenues of about $2,725-$5,450 which translates to about $7-$14/day way above the poverty line of $1.9/day. These compare quite well with the annual average earnings of employees with primary education ($2,386, lower secondary education ($2,897) and upper secondary education ($3,439) [32]. Considering an average household size of 4 persons, the above translate to 1.8–3.7 $/day for smallholder farmers and 1-6-3.2 $/day for educated employees. Smallholders can derive additional revenue from selling parts of the oil palm trees other than FFBs, such as loose fruits and fronds which are valuable for livestock feeds [38]. Investment in palm oil production can and does contribute to poverty alleviation in many African countries fulfilling sustainable development goal (SDG) 1. This contribution and others including SDG 1 and 2 may come with a negative impact on other SDGs if the negative impacts are not addressed such as those in SDG 12 [39]. To address negative impacts of palm oil trade, national principles and action plans are actively being developed in several countries in Africa, as part of the ambitious palm oil development plans (APOI) which aims to guide the design and implementation of a set of regional principles that will reduce deforestation, encourage smallholder production, and improve livelihoods while promoting socio-economic growth [36, 40]. In this study, and recommendations from other studies show that issues such as access to land, access to finance and good management practice are key to achieving sustainable palm oil production while improving livelihoods [41].

The profitability of palm oil investment is dependent on several factors including access to land, access to financing as well as access to milling and good management practices that improves yields. Access to land have been linked to poverty alleviation in many parts of the world but particularly in Africa where several authors including Jayne et al. [42] showed a strong positive relationship between access to land and household income [43]. Our sensitivity analysis shows that, access to land at no cost is the single most important factor in the profitability of palm oil production. Indeed, a key finding is that in all locations, investment in palm oil production is mostly profitable if land is not bought (Fig 5). The severity of the impact of land cost is seen particularly in Lobe where there are loses as soon as you include land cost except for very small gains at less than $500/ha. In Cameroon, many medium- and large-scale oil palm producers are political elites or wealthy businessmen and women engaged in oil palm cultivation as an investment opportunity [12]. This investment is only viable if these people own land but if they buy land, the investment does not necessarily yield good profit. The role of land ownership in profitability is particularly worrying as women are at a disadvantage in the palm oil sector because of lack of land, limited capital and limited access to extension services [44]. Due to ethnic socio-cultural constraints, women as opposed to men do not have easy access to inherited land, for fear that in the future, the land will no longer be the property of the family, should the woman eventually get married. Indeed, land is an important source of security against poverty across the continent and developing world, unequal rights to land put women at a disadvantage, perpetuate poverty, and entrench gender inequality in Africa [43]. Land grabbing, land rights/access and conflicts related to land were one of the top most reported negative impacts on palm oil trade around the world [39]. Currently, Cameroon’s Land Ordinance Laws passed in 1974 present a complexity in which the state administers national lands belonging to Cameroonian citizens, and claims ownership of unregistered, un-titled public lands [45]. According to Ordway et al. [12] most smallholder oil palm farmers (87%) claimed ownership of 100% of the land they were cultivating for oil palm, yet only 5% had a state title. Realistic discussions of poverty alleviation strategies in Africa need to be grounded in the context of land distribution patterns and trends addressing the issue around gender bias associated with land tenure [42].

Aside from land ownership, access to financial resources as recommended by Ordway et al. [12] is an important factor in the profitability of investment in oil palm in Cameroon and probably elsewhere in Africa. Our results show that smallholder farmers can obtain an NPV of between $5,000 and $14,000 when the interest rate in below 7% particularly in Dibombari and Muyuka. However, these numbers drop to less than $4000 in Lobe and Eseka. Interest rate more than 7% does not produce significant profit and produces loss at about 9%. These results are important because previous government schemes and partnerships with micro-finance institutions have provided loans to smallholders at 8–12% interest rates which is above the rate required to make significant profit. The results of our sensitivity analysis reveal that at these interest rates, investment in oil palm plantation may not be necessarily viable and does not yield profit. Government and micro-finance organizations, credit unions and rural development banks should play a role in financing smallholder oil palm at a reasonable interest rate. Several other measures exist to improve profitability not included in this study. For example, oil palm trunks can be used for farming palm weevils (*Rhynchophorus* spp) which are an excellent low-cost source of essential nutrients and are highly prized in the regional market [46]. A single oil palm trunk in 3–4 months can produce 1–4 kg of palm weevil larvae for animal or human consumption, worth $10-$40 [47]. The bulk of the oil palm trunk can also be used for palm wine, mushroom cultivation or energy generation. In addition, the nutrient rich frass arising from weevil farming (i.e., insect waste), along with other oil palm waste, can be used to improve soils and avoid the cost associate with fertilizers. Finally, the hollowed palm logs have further market value as ornamental containers [48].

Milling is an important aspect in improving profitability of smallholder investment in oil palm. Results from this study shows that in all metrics considered, producing and selling CPO performed better that producing and selling FFBs. In addition, most smallholders process their FFBs in artisanal mills with extraction rates of 12–15% compared to 20–23% in agro-industrial mills. To overcome the problems associated with these low-efficiency mills, smallholders are encouraged to either sell their FFBs to agro-industrial mills or use improved mechanized mills. In response to the issue of low extraction rates, UNIDO has invested in four pilot high-efficiency mills aimed to serve groups of smallholders. The pilot mills are located at Green Valley Plantation, Bakingili-Limbe, and Ejagham Oil Palm Cooperative at Mkpot both in the South West Region; BAMSO Common Initiative Group at Sombo, Center Region; and Multipurpose Cooperative Society at Ngie, North West Region [49]. However, for smallholders to exploit the opportunity of using such mills, the incremental benefits that they obtain from the new mills must equal or exceed that of the low-efficiency mills. In addition, smallholders should be educated on the importance of using such mills. An evaluation of the use of the UNIDO mills is needed to understand opportunity and barriers in optimizing benefits from community owned mills. Recent findings also show that smallholder FFB yields have been low in the four production basins due to aging oil palm plantations, use of low-quality planting materials, little or no fertilizer application and other poor management practices [20]. For example, smallholder old oil palm stands around SOCAPALM (Dibombari and Eseka) have averaged 6–7 tons of FFB/ha/year compared to a potential 20 tons of FFB/ha/yr. The productivity of oil palm plantations is highly dependent on the supply of resources (e.g., water, fertilizers and sunlight) and on good plantation management practices [7]. Poor management practices, including incomplete crop recovery (i.e., harvesting all suitable crops) and inadequate agronomic management have been reported as the main factors contributing to FFB yield gaps [15]. Oil palm productivity can be increased through best management practices (improvement of technical culture) and the use of high-quality seeds (some can produce around 35 tons of FFB/ha) [50].

Therefore, widespread use of optimal practices can help meet growing global demand without the need to convert pristine ecosystems into new plantations [7]. In Cameroon, Jelsma et al. [14] reported that smallholder farmers opt for a low-input low-output system for several reasons and that under existing conditions, initiatives such as improving access to finance or availability of good planting materials alone are unlikely to significantly improve the productivity and sustainability of the smallholder oil palm sector. However, in Indonesia, contracts between agro-industrial companies and local communities have significantly contributed to wealth accumulation. Well-designed contracts were found to be important for smallholders to benefit from the oil palm boom [51]. Effective guidance and advice (training) of smallholder farmers leads to better GAPs and productivity [52]. Education and guidance campaigns are needed to increase the awareness on high yield and sustainable oil palm production among independent smallholders. Modifying the spacing of trees within larger plantations can add layers to income generation through enhanced intercropping and increasing ecosystem service provisions [48]. Oil palm intercropping has been shown to improve the livelihoods of smallholder farmers and reduce weeding costs in Ghana and Cameroon as well as buffering their incomes while oil palms come to production [53]. For example, maize, cassava and plantain can be intercropped with oil palms, initially planted around 1 m away from the oil palm trees, but with increasing distances as the palm grows and fronds spread. Four to five years after establishment, oil palm canopy growth shifts intercropping to shade-tolerant species such as climbing peppers. This can provide important additional food crops and revenues in these regions, from about $1,500 per ha (maize) to possibly $9,400 annually (cassava). A mean annual wage of $1,281 is obtained per hectare per household for smallholders practicing intercropping in Cameroon [48]. With a strong institutional arrangement, smallholder oil palm farmers can participate in supply chains on advantageous conditions and substantially increase productivity, thereby contributing to both rural development and land sparing [54].

While agro-industries are efficient in terms of better machinery, processing equipment, technical knowledge and management prerogatives, which translates into better yields and higher extraction efficiency, smallholder or family farming is better in terms of job creation, poverty alleviation and social justice [55]. Due to lack of access to good quality seedlings, use of little inputs, and sale of FFBs to industrial millers, smallholders in Cameroon have low economic returns amounting to about 300 euro/ha/yr at peak production age [56] against 800 to 2,900 euro/ha/yr for an Indonesian smallholder [57]. However, processing FFBs in artisanal mills has a higher value added to the FFBs and provides additional income to smallholder farmers [20].

## 6. Conclusion

Global demand for palm oil has been continuously increasing and this demand has been met by expansion of oil palm plantations. In Cameroon as other parts of Africa, smallholder growers are increasingly occupying more cultivated oil palm area than agro-industrial companies. Sustainable production of palm oil depends on improving financial profitability without necessarily increasing land area cultivated. We found that investment in oil palm plantations is a profitable business for smallholders in all locations considered in this study. However, several factors affect the financial viability of smallholders investing in palm oil trade with the cost of land being the most significant. Smallholders make more money when they process their FFBs and sell the CPO than sell them to intermediaries or agro-industrial mills. Favourable interest rates generally below 7% are needed to render investments by smallholders profitable. This is an important aspect as about 33% of farmers obtain their capital through bank and cooperatives loans [44]. Improving milling efficiency to increase CPO yields, use of good agricultural practices to increase FFB yields, use of by-products and wastes are measures that smallholders can use to increase their revenues from palm oil trade.

Although the data used in this study are from four of the seven oil palm production basins in Cameroon, the results from the sensitivity analysis show the range of values and parameters that have significant impacts on profitability for smallholders and can be generalized in the country. Similar studies should be carried out for other commodities to compare the results in order to inform potential investors on which crops are more profitable and why they should choose to invest in one commodity over the other. Policy makers can also use these results to design policies to support revenue generation while reducing deforestation in the sector.

## Supporting information

S1 File These are the metrics for evaluating the financial viability. (DOCX)

S1 Table NPV ($) for different percentage changes in CPO processing cost, field management cost, and field yield in different locations. (DOCX)
