# Supplementary material for: Does investment in palm oil trade alleviate smallholders from poverty in Africa? Investigating profitability from a biodiversity hotspot, Cameroon
Source: PLoS One. 2021 Sep 1;16(9):e0256498. doi: 10.1371/journal.pone.0256498 (PMC8409616; doi:10.1371/journal.pone.0256498)
Supplement: S1 File — (DOCX) [file pone.0256498.s001.docx]

**S1. Metrics for evaluating the financial viability**

**S1.1 Net Present Value**

The Net present value (NPV) is the difference between the present value of cash inflows and the present value of cash outflows over a period of time. A target rate of return or interest rate is set which is used to discount the net cash inflows from a project. Any project or investment with a negative NPV should be avoided while implementation is justified if the NPV is positive or equal to zero. The formula for calculating NPV is [58]:

$\text{NPV=}\sum_{\text{t=1}}^{\text{t=T}} \frac{\text{R}_{\text{t}}}{\text{(1+d)}^{\text{t}}}\text{-}\sum_{\text{t=1}}^{\text{t=T}} \frac{\text{C}_{\text{t}}}{\text{(1+d)}^{\text{t}}}\text{-}\text{ }\text{C}_{\text{0}}$ (S1.1)

where, NPV ($), R_t_ is the annual revenue in year t ($), C_t_ is the annual cost in year t ($), C_0_ is the cost in year 0 ($), t is the duration of the project (years), d is the discount rate (%).

**S1.2 Internal Rate of Return**

The internal rate of return (IRR) is an indicator of the efficiency or quality of an investment. IRR is the interest rate at which revenues are equal to costs, i.e. the rate of return that would deliver an NPV equal to 0. Theoretically speaking, a project is worth investing in from a private viewpoint if the IRR is higher than the market interest rate [59, 35]. IRR is calculated using Eqn. S1.2:

$\text{NPV=}\sum_{\text{t=1}}^{\text{t=T}} \frac{\text{R}_{\text{t}}}{\text{(1+IRR)}^{\text{t}}}\text{-}\sum_{\text{t=1}}^{\text{t=T}} \frac{\text{C}_{\text{t}}}{\left( \text{1+IRR} \right)^{\text{t}}}\text{-}{\text{ }\text{C}}_{\text{0}}\text{=0}$ (S1.2)

Where, IRR is the internal rate of return (%).

**S1.3 Benefit-Cost Ratio**

Benefit-Cost Ratio (BCR) is the benefit received per unit cost. BCR can be used to determine the most cost-efficient choice of development [60]. If BCR > 1, the project is justified on economic grounds, and cost-effectiveness increases with the value of BCR. The BCR is calculated as follows:

$BCR=\frac{\sum_{t=1}^{t=T} \frac{R_{t}}{\left( 1+d \right)^{t}}}{\left( C_{0}+\sum_{t=1}^{t=T} \frac{C_{t}}{\left( 1+d \right)^{t}} \right)}$ (S1.3)

Where all variables are as in Eqn. S1.2.

**S1.4 Payback Period**

The payback period (PP) is the period of time (usually in years) required for the profit or other benefits of an investment to equal the cost of the investment [35]. The shorter the PP, the more desirable the project is because the recovered funds can be used for future investments. PP does not however consider the differences in the timing of cash flows. PP is calculated as the ratio of the cost of investment divided by the annual cash inflows (Eqn. S1.4).

$\text{PP=}\frac{\text{C}_{\text{0}}}{\text{R}_{\text{t}}}$ (S1.4)

**References**

35. Newnan DG, Lavelle JP, Eschenbach TG. Essentials of engineering economic analysis. Second Ed., Oxford University Press, Oxford, UK, 2002.

58. Duffy A, Rogers M, Ayompe L. Renewable Energy and Energy Efficiency: Assessment of Projects and Policies. Wiley, London, 2015.

59. Meggison WL. Corporate Finance Theory. Addison-Wesley, Reading, MA, 1997, 506 pp.

60. Turner RK, Van den Bergh JCJM, Soderqvist T, Barendregt A, Van der Straaten, J, Maltby E, Van Ierland EC. Ecological economic analysis of wetlands: scientific integration for management and policy. Ecological Economics 2000; 35, 7-23. DOI: 10.1016/S0921-8009(00)00164-6
