# Supplementary material for: Does investment in palm oil trade alleviate smallholders from poverty in Africa? Investigating profitability from a biodiversity hotspot, Cameroon
Source: PLoS One. 2021 Sep 1;16(9):e0256498. doi: 10.1371/journal.pone.0256498 (PMC8409616; doi:10.1371/journal.pone.0256498)
Supplement: S1 Table — (DOCX) [file pone.0256498.s002.docx]

**S1 Table.** NPV ($) for different percentage changes in CPO processing cost, field management cost, and field yield in different locations.

| **Location** | **Percentage**  **change (%)** | **CPO processing cost** | | | **Field management cost** | | | **FFB yield** | | |
| --- | --- | --- | --- | --- | --- | --- | --- | --- | --- | --- |
|  |  | ***FFB(i)*** | ***FFB(a)*** | ***CPO*** | ***FFB(i)*** | ***FFB(a)*** | ***CPO*** | ***FFB(i)*** | ***FFB(a)*** | ***CPO*** |
| Dibombari | -80 | 130 | 545 | ***5,692*** | ***4,671*** | ***5,085*** | ***7,462*** | -3,520 | -3,438 | -2,962 |
|  | -60 | 130 | 545 | ***4,999*** | ***3,515*** | ***3,950*** | ***6,327*** | -2,608 | -2,442 | -1,491 |
|  | -40 | 130 | 545 | ***4,307*** | ***2,400*** | ***2,815*** | ***5,192*** | -1,695 | -1,446 | -20 |
|  | -20 | 130 | 545 | ***3,614*** | ***1,265*** | ***1,680*** | ***4,057*** | -783 | -451 | ***1,451*** |
|  | **0** | 130 | 545 | ***2,921*** | ***130*** | ***545*** | ***2,921*** | ***130*** | ***545*** | ***2,921*** |
|  | 20 | 130 | 545 | ***2,229*** | -1,005 | -590 | ***1,786*** | ***1,043*** | ***1,540*** | ***4,392*** |
|  | 40 | 130 | 545 | ***1,536*** | -2,140 | -1,725 | ***651*** | ***1,955*** | ***2,516*** | ***5,863*** |
|  | 60 | 130 | 545 | ***844*** | -3,275 | -2,861 | -484 | ***2,868*** | ***3,532*** | ***7,334*** |
|  | 80 | 130 | 545 | **151** | -4,410 | -3,996 | -1,619 | ***3,781*** | ***4,527*** | ***8,805*** |
|  | 100 | 130 | 545 | -542 | -5,545 | -5,131 | -2,754 | ***4,693*** | ***5,523*** | ***10,276*** |
| Eseka | -80 | -265 | 545 | ***3,347*** | ***4,275*** | ***5,085*** | ***5,054*** | -3,599 | -3,438 | -3,444 |
|  | -60 | -265 | 545 | ***2,639*** | ***3,140*** | ***3,950*** | ***3,919*** | -2,766 | -2,442 | -2,454 |
|  | -40 | -265 | 545 | ***1,930*** | ***2,005*** | ***2,815*** | ***2,784*** | -1,912 | -1,446 | -1,465 |
|  | -20 | -265 | 545 | ***1,222*** | ***870*** | ***1,680*** | ***1,649*** | -1,099 | -451 | -476 |
|  | **0** | -265 | 545 | ***514*** | -265 | ***545*** | ***514*** | -265 | ***545*** | ***514*** |
|  | 20 | -265 | 545 | -195 | -1,400 | -590 | -621 | ***568*** | ***1,540*** | ***1,503*** |
|  | 40 | -265 | 545 | -903 | -2,535 | -1,725 | -1,757 | ***1,402*** | ***2,536*** | ***2,492*** |
|  | 60 | -265 | 545 | -1,611 | -3,670 | -2,861 | -2,892 | ***2,236*** | ***3,532*** | ***3,482*** |
|  | 80 | -265 | 545 | -2,320 | -4,806 | -3,996 | -4,027 | ***3,069*** | ***4,527*** | ***4,471*** |
|  | 100 | -265 | 545 | -3,028 | -5,941 | -5,131 | -5,162 | ***3,903*** | ***5,523*** | ***5,460*** |
| Muyuka | -80 | -46 | 804 | ***6,001*** | ***4,495*** | ***5,344*** | ***8,393*** | -3,556 | -3,386 | -2,776 |
|  | -60 | -46 | 804 | ***5,464*** | ***3,360*** | ***4,209*** | ***7,258*** | -2,678 | -2,338 | -1,119 |
|  | -40 | -46 | 804 | ***4,927*** | ***2,224*** | ***3,074*** | ***6,123*** | -1,801 | -1,291 | ***538*** |
|  | -20 | -46 | 804 | ***4,390*** | ***1,089*** | ***1,939*** | ***4,988*** | -923 | -243 | ***2,195*** |
|  | **0** | -46 | 804 | ***3,853*** | -46 | ***804*** | ***3,853*** | -46 | ***804*** | ***3,853*** |
|  | 20 | -46 | 804 | ***3,316*** | -1,181 | -331 | ***2,718*** | ***812*** | ***1,851*** | ***5,510*** |
|  | 40 | -46 | 804 | ***2,779*** | -2,316 | -1,466 | ***1,582*** | ***1,709*** | ***2,899*** | ***7,167*** |
|  | 60 | -46 | 804 | ***2,242*** | -3,451 | -2,601 | ***447*** | ***2,587*** | ***3,946*** | ***8,824*** |
|  | 80 | -46 | 804 | ***1,705*** | -4,586 | -3,736 | -688 | ***3,464*** | ***4,994*** | ***10,481*** |
|  | 100 | -46 | 804 | ***1,168*** | -5,721 | -4,872 | -1,823 | ***4,342*** | ***6,041*** | ***12,138*** |
| Lobe | -80 | -719 | -233 | ***3,148*** | ***3,822*** | ***4,308*** | ***5,217*** | -3,690 | -3,593 | -3,411 |
|  | -60 | -719 | -233 | ***2,530*** | ***2,686*** | ***3,173*** | ***4,082*** | -2,947 | -2,753 | -2,389 |
|  | -40 | -719 | -233 | ***1,912*** | ***1,551*** | ***2,037*** | ***2,947*** | -2,205 | -1,913 | -1,367 |
|  | -20 | -719 | -233 | ***1,295*** | ***416*** | ***902*** | ***1,812*** | -1,462 | -1,073 | -345 |
|  | **0** | -719 | -233 | ***677*** | -719 | -233 | ***677*** | -719 | -233 | ***677*** |
|  | 20 | -719 | -233 | ***59*** | -1,854 | -1,368 | -458 | ***24*** | ***607*** | ***1,699*** |
|  | 40 | -719 | -233 | -558 | -2,989 | -2,503 | -1,593 | ***767*** | ***1,447*** | ***2,721*** |
|  | 60 | -719 | -233 | -1,176 | -4,124 | -3,638 | -2,728 | ***1,510*** | ***2,287*** | ***3,743*** |
|  | 80 | -719 | -233 | -1,794 | -5,259 | -4,773 | -3,863 | ***2,252*** | ***3,128*** | ***4,765*** |
|  | 100 | -719 | -233 | -2,411 | -6,394 | -5,908 | -4,999 | ***2,995*** | ***3,968*** | ***5,787*** |

FFB(i): FFB sale to intermediaries; FFB(a): FFB sale to agro-industrial mills; CPO: crude palm oil sale
